# Sagittal Jaw Relationship of Different Types of Cleft and Non-cleft Individuals

**DOI:** 10.3389/fped.2021.651951

**Published:** 2021-05-05

**Authors:** Mohammad Khursheed Alam, Ahmed Ali Alfawzan, Sanjida Haque, Pooi Ling Mok, Anand Marya, Adith Venugopal, Nafij Bin Jamayet, Ammar A. Siddiqui

**Affiliations:** ^1^Orthodontic Division, Department of Preventive Dental Science, College of Dentistry, Jouf University, Sakaka, Saudi Arabia; ^2^Department of Preventive Dentistry, College of Dentistry in Ar Rass, Qassim University, Ar Rass, Saudi Arabia; ^3^Queens Hospital (pvt) Limited, Jashore, Bangladesh; ^4^Department of Clinical Laboratory Sciences, College of Applied Medical Sciences, Jouf University, Sakaka, Saudi Arabia; ^5^Department of Biomedical Sciences, Faculty of Medicine and Health Sciences, Universiti Putra Malaysia, Serdang, Malaysia; ^6^Section of Orthodontics, University of Puthisastra, Phnom Penh, Cambodia; ^7^Department of Orthodontics, Saveetha Dental College, Saveetha Institute of Medical and Technical Sciences, Saveetha University, Chennai, India; ^8^Division of Clinical Dentistry (Prosthodontics), School of Dentistry, International Medical University, Kuala Lumpur, Malaysia; ^9^Department of Community Dentistry, College of Dentistry, Bakhtawar Amin Medical and Dental College, Multan, Pakistan

**Keywords:** sagittal jaw relationship, cleft lip and palate, cephalometric analysis, wits appraisal, SNA angle, SNB angle, ANB angle, artificial intelligence

## Abstract

To investigate whether the craniofacial sagittal jaw relationship in patients with non-syndromic cleft differed from non-cleft (NC) individuals by artificial intelligence (A.I.)-driven lateral cephalometric (Late. Ceph.) analysis. The study group comprised 123 subjects with different types of clefts including 29 = BCLP (bilateral cleft lip and palate), 41 = UCLP (unilateral cleft lip and palate), 9 = UCLA (unilateral cleft lip and alveolus), 13 = UCL (unilateral cleft lip) and NC = 31. The mean age was 14.77 years. SNA, SNB, ANB angle and Wits appraisal was measured in lateral cephalogram using a new innovative A.I driven Webceph software. Two-way ANOVA and multiple-comparison statistics tests were applied to see the differences between gender and among different types of clefts vs. NC individuals. A significant decrease (*p* < 0.005) in SNA, ANB, Wits appraisal was observed in different types of clefts vs. NC individuals. SNB (*p* > 0.005) showed insignificant variables in relation to type of clefts. No significant difference was also found in terms of gender in relation to any type of clefts and NC group. The present study advocates a decrease in sagittal development (SNA, ANB and Wits appraisal) in different types of cleft compared to NC individuals.

## Introduction

Cleft lip and palate; the second most common developmental abnormalities which extant during birth by presenting anatomical alteration of the lip and/or palate (1). This craniofacial malformation is caused by the effects of both genetic and environmental factors such as maternal smoking and alcohol consumption, stress, viral infection during the first 8 weeks of pregnancy, teratogenic drug etc. (2).

The treatment of cleft lip and palate (CLP) is multidisciplinary where the only purpose is to restore the functional and aesthetic value. Many beneficial approaches have been carried out previously such as: dental arch relationship (3), arch dimension (4), tooth size morphometry (5) and craniofacial morphology (6) to evaluate the outcome of CLP patients.

Atypical sagittal growth of maxilla is a common manifestation in patients with UCLP; acknowledged by many researchers yet all of them came to an understanding that the growth and direction of the jaw utterly influenced by the earlier treatment protocol such as time and techniques of primary surgeries (7–12). The detrimental effect of palatoplasty on sagittal growth of maxilla has been widely documented in literature (9) but whether cheiloplasty impedes the growth is still in controversy (8, 13). A number of researchers found that cheiloplasty has an effect on maxillary incisors, alveolar bone and development of maxilla as well whether some researchers claimed no effect on maxillary development (7, 11, 12, 14, 15).

The craniofacial characteristics of CLP can be assessed from cephalogram (16) and cone-beam computed tomography (17) as well. Abundant cephalometric studies have been done on CLP yet restricted to three-dimensionally. Artificial intelligence (A.I.) into dentistry, especially in cleft research is still a new-fangled and robust technique (18–21).

Use of A.I driven Webceph software for the measurements of all variables in cephalogram were the particularities from previous study which is more accurate, precise, robust and reliable compared to manual measurements (18–21).

For the first time, the present study tried to disclose the sagittal development of Saudi Arabian CLP patients, by A. I driven lateral cephalometric analysis and compared it with the non-cleft (NC) individuals and also compared in relation to gender and types of cleft.

## Materials and Methods

This retrospective study was limited to 123 individuals including 29 bilateral cleft lip and palate (BCLP), 41 unilateral cleft lip and palate (UCLP), 13 unilateral cleft lip UCL, 9 unilateral cleft lip and alveolus (UCLA) and 31 NC individuals with the average age of 13.29 [3.52] (NC), 14.07 [4.73] (BCLP), 14.32 [4.46] (UCLP), 12.78 [4.09] (UCLA), and 13.31 [4.46] (UCL) years, respectively. All the data (medical records and X-rays) of this study were collected from Saudi Board of Dental Residents and approved by the Ethical Committee of Al Rass Dental Research Center, Qassim University (DRC/009FA/20). Non-syndromic cleft individuals with good-quality x-ray images were included whereas any history of craniofacial surgery, bone grafting and orthodontic treatment was excluded from the study. Same age group of healthy non cleft individuals were also included as the control group. Convenient sampling has been done without randomization.

Digital Lateral Cephalogram X-rays were used to measure four different cephalometric parameters (SNA, SNA, ANB and Wits appraisal) to investigate sagittal characteristics of 123 individuals of cleft and non-cleft group. All the cephalometric parameters were measured by one examiner using automated (20, 21) A.I.-driven Webceph software (South Korea). The angular and linear measurements used in this study are detailed in Table 1.

**Table 1:** The angular and linear cephalometric measurements with description.

**Measurements**	**Description**
SNA (The sella-nasion-A point)	An angle relates to the antero-posterior position of the maxillary apical base to a line passing through the anterior cranial base.
SNB (The sella-nasion-B point)	An angle relates to the antero-posterior position of the mandibular apical base to a line passing through the anterior cranial base.
ANB (A point- nasion-B Point)	An angle relates to the antero-posterior relationship of the mandible to the maxilla.
Wits appraisal	A linear cephalometric analytic obtained by projecting straight lines from the A and B points, respectively, unto the functional occlusal plane at 90° and measuring the horizontal distance form point AO to BO

### Statistical Analysis

Intra-class correlation coefficients were executed to assess the intra-examiner reliability with 20 randomly selected x-rays after 2-weeks interval and found 0.916–0.990 for all measurements which indicates excellent reliability. A two-way ANOVA examination was utilized for gender orientation, types of cleft and gender types of cleft. SPSS 24 (SPSS Inc., Chicago, IL, United States) was used to scrutinize all the data and *p*-value was set as <0.05.

## Results

### Results of SNA Angle

The mean SNA angle of NC, UCLP, BCLP, UCL and UCLA were 79.795°, 76.158°, 74.953°, 79.443°, and 78.042°, respectively. UCLP (*p* < 0.001) and BCLP (*p* < 0.001) subjects had significantly smaller SNA angles compared to NC subjects. Significant difference also observed in two groups. They are BCLP vs. UCL (*p*-value 0.002) and BCLP vs. UCLA (*p*-value 0.001). However, no significant differences were found in terms of gender.

Table 2 shows the detailed results of SNA angle in relation to gender and types of cleft and NC.

**Table 2:** Sagittal analysis—SNA: Gender, Types of Cleft and Gender times types of cleft two-way ANOVA analysis results.

**Gender**	**Type**	**Mean**	**SD**	**Cleft type**	**Mean**	**Multiple comparison**			**MD**	**SE**	***p*-value**	**95% CI**	
												**Lower bound**	**Upper bound**
Male	NC	79.296	3.306	NC	79.795	NC	vs	BCLP	4.841^*^	1.108	0.000	2.646	7.036
	BCLP	74.654	4.346	BCLP	74.953		vs	UCLP	3.637^*^	0.961	0.000	1.733	5.541
	UCLP	76.267	4.751	UCLP	76.158		vs	UCL	0.352	1.335	0.792	−2.293	2.997
	UCL	77.709	3.887	UCL	79.443		vs	UCLA	1.752	1.598	0.275	−1.414	4.918
	UCLA	78.542	3.424	UCLA	78.042	BCLP	vs	UCLP	−1.204	1.046	0.252	−3.277	0.869
	Total	76.735	4.429				vs	UCL	−4.489^*^	1.398	0.002	−7.258	−1.720
Female	NC	80.294	3.641				vs	UCLA	−7.026^*^	1.704	0.001	−11.905	−2.146
	BCLP	75.253	2.303			UCLP	vs	UCL	−3.285^*^	1.284	0.012	−5.830	−0.740
	UCLP	76.049	4.170				vs	UCLA	−1.885	1.556	0.228	−4.968	1.198
	UCL	81.177	5.072			UCL	vs	UCLA	1.400	1.811	0.441	−2.188	4.988
	UCLA	77.543	1.139										
	Total	77.920	4.339										
Total	NC	79.843	3.473			*p*-value	PES						
	BCLP	74.819	3.859		Gender	0.387	0.007						
	UCLP	76.160	4.423		Cleft Type	0.000	0.192						
	UCL	79.309	4.638		Gender ^*^ Cleft Type	0.654	0.021						
	UCLA	78.209	2.811										
	Total	77.255	4.412										

*SD, standard deviation; MD, mean difference; SE, standard error; CI, confidence interval; PES, partial eta square*.

* *= Significant difference*.

### Results of SNB Angle

The mean SNB angle of NC, UCLP, BCLP, UCL, and UCLA were 76.017°, 76.230°, 75.496°, 76.632°, and 75.917°, respectively. No significant differences found in terms of gender and types of cleft (Table 3).

**Table 3:** Sagittal analysis—SNB: Gender, Types of Cleft and Gender times types of cleft two-way ANOVA analysis results.

**Gender**	**Type**	**Mean**	**SD**	**Cleft type**	**Mean**	**Multiple comparison**			**MD**	**SE**	***p*-value**	**95% CI**	
												**Lower bound**	**Upper bound**
Male	NC	75.739	3.548	NC	76.017	NC	vs	BCLP	0.521	1.139	0.648	−1.736	2.777
	BCLP	74.190	5.326	BCLP	75.496		vs	UCLP	−0.214	0.988	0.829	−2.171	1.744
	UCLP	77.032	3.368	UCLP	76.230		vs	UCL	−0.615	1.372	0.655	−3.334	2.104
	UCL	74.916	4.630	UCL	76.632		vs	UCLA	0.100	1.643	0.951	−3.155	3.355
	UCLA	76.490	5.171	UCLA	75.917	BCLP	vs	UCLP	−0.734	1.076	0.496	−2.866	1.397
	Total	75.643	4.395				vs	UCL	−1.136	1.437	0.431	−3.983	1.711
Female	NC	76.295	3.111				vs	UCLA	−0.421	1.697	0.805	−3.783	2.942
	BCLP	76.803	5.139			UCLP	vs	UCL	−0.402	1.321	0.762	−3.018	2.215
	UCLP	75.429	3.497				vs	UCLA	0.314	1.600	0.845	−2.856	3.484
	UCL	78.348	4.859			UCL	vs	UCLA	0.715	1.862	0.702	−2.974	4.405
	UCLA	75.343	3.585										
	Total	76.225	3.788										
Total	NC	76.044	3.271			*p*-value	PES						
	BCLP	74.910	5.318		Gender	0.399	0.006						
	UCLP	76.250	3.484		Cleft Type	0.943	0.007						
	UCL	76.500	4.871		Gender ^*^ Cleft Type	0.201	0.051						
	UCLA	76.108	4.500										
	Total	75.898	4.133										

*SD, standard deviation; MD, mean difference; SE, standard error; CI, confidence interval; PES, partial eta square*.

* *= Significant difference*.

### Results of ANB Angle

The mean ANB angle of NC, UCLP, BCLP, UCL and UCLA were 3.773°, −0.073°, −0.543°, 2.811°, and 2.128°, respectively. UCLP (*p*-value <0.001) and BCLP (*p*-value <0.001) subjects had significantly smaller ANB angles compared to NC subjects. However, no significant differences were found in terms of gender (Table 4).

**Table 4:** Sagittal analysis—ANB: Gender, Types of Cleft and Gender times types of cleft two-way ANOVA analysis results.

**Gender**	**Type**	**Mean**	**SD**	**Cleft type**	**Mean**	**Multiple comparison**			**MD**	**SE**	***p*-value**	**95% CI**	
												**Lower bound**	**Upper bound**
Male	NC	3.556	1.800	NC	3.778	NC	vs	BCLP	4.321^*^	1.047	0.000	2.247	6.396
	BCLP	0.465	4.604	BCLP	−0.543		vs	UCLP	3.851^*^	0.908	0.000	2.051	5.650
	UCLP	−0.764	3.497	UCLP	−0.073		vs	UCL	0.967	1.262	0.445	−1.533	3.467
	UCL	2.794	5.857	UCL	2.811		vs	UCLA	1.650	1.511	0.277	−1.342	4.643
	UCLA	2.052	4.138	UCLA	2.128	BCLP	vs	UCLP	−0.470	0.989	0.635	−2.430	1.489
	Total	1.092	4.190				vs	UCL	−3.355^*^	1.321	0.012	−5.972	−0.737
Female	NC	3.999	2.683				vs	UCLA	−2.671	1.561	0.090	−5.762	0.421
	BCLP	−1.551	5.545			UCLP	vs	UCL	−2.884^*^	1.214	0.019	−5.290	−0.479
	UCLP	0.619	3.004				vs	UCLA	−2.200	1.471	0.137	−5.115	0.714
	UCL	2.828	4.256			UCL	vs	UCLA	0.684	1.712	0.690	−2.708	4.076
	UCLA	2.203	4.580										
	Total	1.695	3.982										
Total	NC	3.799	2.301			*p*-value	PES						
	BCLP	−0.091	4.865		Gender	0.999	.000						
	UCLP	−0.090	3.300		Cleft Type	0.000	0.188						
	UCL	2.810	4.970		Gender ^*^ Cleft Type	0.562	0.026						
	UCLA	2.102	3.994										
	Total	1.357	4.094										

*SD, standard deviation; MD, mean difference; SE, standard error; CI, confidence interval; PES, partial eta square*.

* *= Significant difference*.

### Results of Wits Appraisal

The mean dimensions of NC, UCLP, BCLP, UCL and UCLA were 0.474, −2.599, −3.216, −0.635, and −1.068 mm, respectively. UCLP (*p*-value 0.003) and BCLP (*p*-value 0.002) subjects had significantly smaller Wits appraisal compared to NC subjects. However, no significant differences were found in terms of gender.

**Figure 1:**
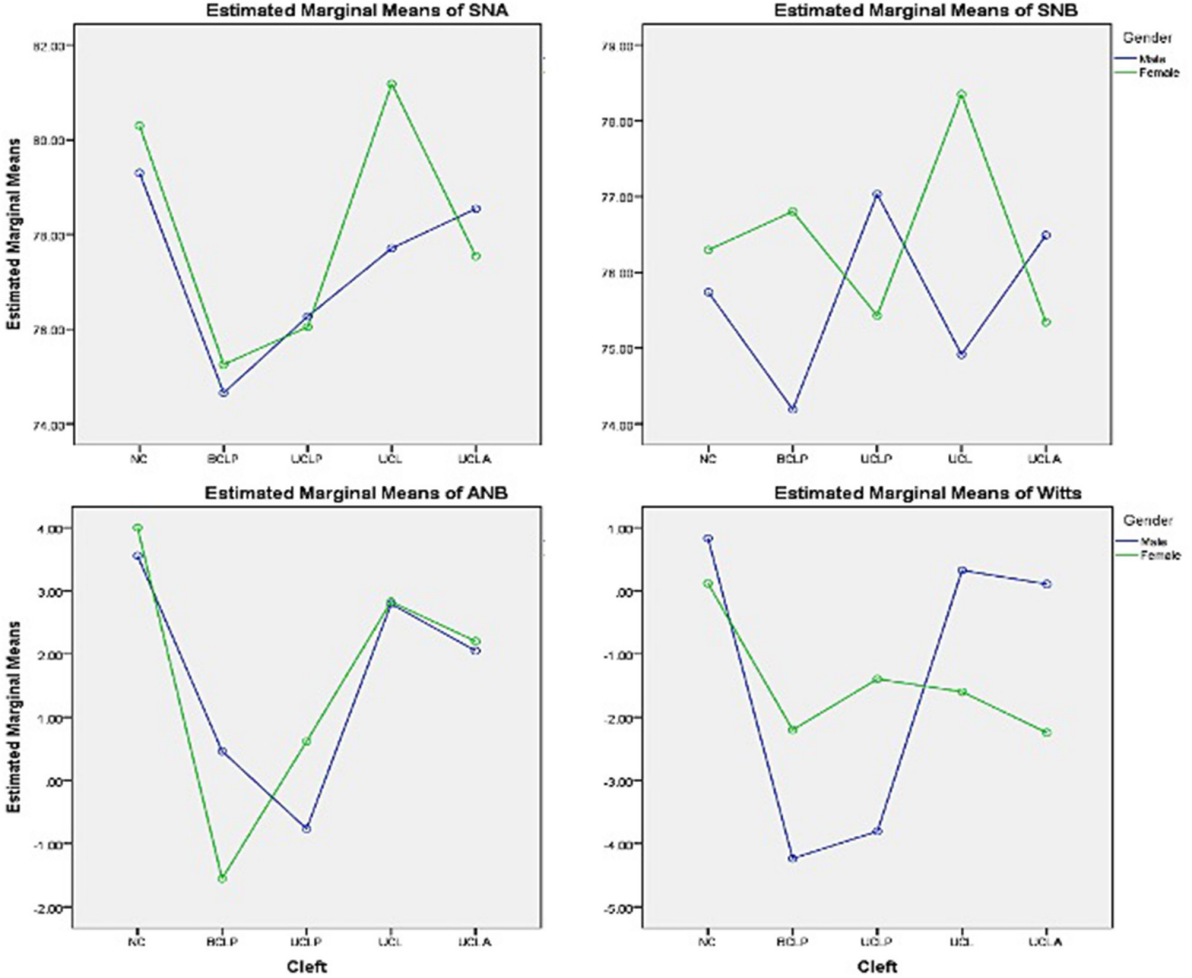
Profile plot of the estimated marginal means of types of cleft and gender*types of cleft.

Table 5 shows the detailed results of Wits appraisal in relation to gender and types of cleft and NC. The profile plot of estimated marginal means of types of cleft and gender^*^types of cleft has been shown in Figure 1.

**Table 5:** Sagittal analysis—Witts analysis: Gender, Types of Cleft and Gender times types of cleft two-way ANOVA analysis results.

**Gender**	**Type**	**Mean**	**SD**	**Cleft type**	**Mean**	**Multiple comparison**			**MD**	**SE**	***p*-value**	**95% CI**	
												**Lower bound**	**Upper bound**
Male	NC	0.831	2.769	NC	0.474	NC	vs	BCLP	3.690^*^	1.184	0.002	1.344	6.036
	BCLP	−4.233	5.479	BCLP	−3.216		vs	UCLP	3.073^*^	1.027	0.003	1.038	5.108
	UCLP	−3.804	3.938	UCLP	−2.599		vs	UCL	1.110	1.427	0.438	−1.717	3.937
	UCL	0.326	5.514	UCL	−0.635		vs	UCLA	1.543	1.708	0.368	−1.842	4.927
	UCLA	0.107	5.424	UCLA	−1.068	BCLP	vs	UCLP	−0.617	1.119	0.582	−2.833	1.599
	Total	−2.235	4.979				vs	UCL	−2.581	1.494	0.087	−5.541	.379
Female	NC	0.117	3.678				vs	UCLA	−2.148	1.765	0.226	−5.644	1.348
	BCLP	−2.199	5.031			UCLP	vs	UCL	−1.964	1.373	0.155	−4.684	0.756
	UCLP	−1.395	3.878				vs	UCLA	−1.531	1.663	0.359	−4.826	1.765
	UCL	−1.597	3.169			UCL	vs	UCLA	0.433	1.936	0.823	−3.403	4.268
	UCLA	−2.243	3.425										
	Total	−1.107	3.873										
Total	NC	0.440	3.266			*p*-value	PES						
	BCLP	−3.672	5.350		Gender	0.909	0.000						
	UCLP	−2.629	4.048		Cleft type	0.013	0.106						
	UCL	−0.562	4.515		Gender ^*^ cleft type	0.274	0.044						
	UCLA	−0.677	4.764										
	Total	−1.740	4.544										

*SD, standard deviation; MD, mean difference; SE, standard error; CI, confidence interval; PES, partial eta square*.

* * = Significant difference*.

## Discussion

The prime objective of this paper was to find out and compare the sagittal development among different types of Saudi CLP patients with NC group and also compare it in relation to gender and types of clefts. Even though several researchers were researched on different issues of CLP patients, very few researchers were described on sagittal development of CLP patients. However, no study was documented previously in Saudi population about sagittal development. For the first time, the present work studied the sagittal development among Saudi population exclusively.

In this present study, we measured SNA, SNB, ANB and Wits appraisal of non-syndromic Saudi CLP patients where all the patients completed their primary surgeries (lip surgery and palate surgery) by the first 2 years of life. The maxillofacial growth of a CLP patient is quite convoluted; affected by both congenital and postnatal treatment factors (3). Primary surgeries limited the maxillary growth which leads to maxillary hypoplasia and finally toward Class III malocclusion reported previously (22).

Poor maxillary growth of CLP patients is one of the chief apprehensions of orthodontists for the corrections of the dento-facial discrepancies especially during the period of early adolescence (7). The outcome of the current study may help and support the surgeon to bring out the proper primary surgical techniques and also to the orthodontist to make better treatment plans for CLP patients. The patient who exhibited smaller SNA, ANB and Wits appraisal specifies the poor maxillary growth may perhaps have need of orthognathic surgery in future for the complete correction of dento-facial divergences (7, 23). Wits appraisal is a supreme linear cephalometric parameter which is used to evaluate and assist an additional information with ANB angle for the assessment of skeletal base discrepancies (24).

In our study we found significant reduction of SNA, ANB and Wits appraisal in cleft individuals compared NC individuals which indicated the maxillofacial growth deficiency. However, SNB was an insignificant variable which is a respectable evidence that cleft does not affect on mandibular growth and coincides with one of the earlier studies (25).

The results of current study also match with the outcome of (26) who assessed 45 lateral cephalograms including both cleft and NC individuals reported significantly decreased SNA and ANB between cleft and NC groups and also did not found any significant difference in SNB angle.

An observational study by Holts et al. (27) using German UCLP, BCLP and NC group showed reduced SNA and ANB angle with minor Class III malocclusion in both UCLP and BCLP groups.

Haque et al. (7) put the idea that Modified Millard techniques of Cheiloplasty and Bardach technique of palatoplasty had noticeable negative effect on anterior segment of maxillae especially on inter canine width by studying maxillary arch dimension of Malaysian UCLP subjects. After analyzing the cephalograms of operated Japanese UCLP subjects, Alam et al. (6, 10) also found altered craniofacial morphology in relation to both postnatal treatment factors and congenital factors.

Lisson et al. (28) compared two centers of German children with BCLP ranging from 10 to 18 years in age: center 1 showed significant reduction of ANB and Wits appraisal and center 2 showed significant reduction of ANB and Wits appraisal and significant increase of SNB angle. The outcome of their study revealed underdeveloped maxilla in both centers even though the time and sequence of palatopasty was the main difference between the centers.

The present study also paid attention to gender disparities with sagittal jaw relationships, however did not get any significant difference. In an earlier Saudi cleft study by Alam and Alfawzan (20) on dental characteristics, did not find any significant relation with gender. Similar findings also reported in Bangladeshi UCLP children (11). In contrast, interestingly Arshad et al. (12) reported significant gender disparities in relation to treatment outcome among Pakistani UCLP children. This different result among different populations indicates the racial bias of UCLP subjects.

Although the present study has reached its aim, however, there were some unavoidable limitations. Because of insufficient data from other centers, this study was conducted from a single center. In a future study we plan to do a multi-center study after collection of sufficient data, especially on UCL and UCLA sample from other centers.

## Conclusion

In this present study, we observed,

- significant reduction of SNA angle in UCLP, BCLP, UCL and UCLA; ANB angle and Wits appraisal in UCLP and BCLP compared to NC group.
- no significant difference in SNB angle in any cleft and NC group.
- no significant gender inequalities in relation to any type of CLP and NC group.

## Data Availability Statement

The original contributions presented in the study are included in the article/supplementary material, further inquiries can be directed to the corresponding author/s.

## Ethics Statement

The studies involving human participants were reviewed and approved by the Ethical Committee of Al Rass Dental Research Center, Qassim University (DRC/009FA/20). Written informed consent to participate in this study was provided by the participants' legal guardian/next of kin.

## Author Contributions

All authors listed have made a substantial, direct and intellectual contribution to the work, and approved it for publication.

## Conflict of Interest

The authors declare that the research was conducted in the absence of any commercial or financial relationships that could be construed as a potential conflict of interest.
